# The Law and the Doctor

**Published:** 1901-03

**Authors:** 


### *EDITORIAL NOTES AND COMMENTS.*

The Business office of THE JOURNAL-RECORD is No. 64 Marietta Street.

The Editorial office is 318-319-320 The Prudential.

Address all Business communications to Dr. M. B. Hutchins, Mgr.

Make remittances payable to THE ATLANTA JOURNAL-RECORD OF MEDICINE.

On matters pertaining to the Editorial and Original Communications address Dr. Bernard Wolff, Atlanta.

Reprints of original articles will be furnished at cost price. Requests for the same should always be made *on the manuscript.*

We will present, post-paid, on request, to each contributor of an original article, twenty (20) marked copies of THE JOURNAL-RECORD containing such article.

### NOTICE TO SUBSCRIBERS.

**Those of you who have not paid your subscription account will probably hear from a collection agency soon. We desire to explain that no offense is meant by our placing these accounts for collection. It is simply a matter of convenience. We cannot give the accounts as much attention as an agency can, hence are compelled to employ the agency.**

### THE LAW AND THE DOCTOR.

A PHILADELPHIA judge has recently fined a doctor $10 for contempt of court in failing to answer a summons to appear as a witness. The doctor pleaded in extenuation the necessity of his attendance upon a desperately ill patient. The conduct of the judge scarcely requires comment as regards his lack of those qualities so essential to one in his position—judgment and discretion. The instance brings up the question of precedence of duty and obligation, one which so often disturbs the harmonious relations of the medical and legal professions. The physician's duty to his patient is with him *suprema lex*, but the law arrogantly demands immediate and absolute obedience and recognizes no consideration save death as taking precedence over it. It prates of its majesty, of its divine function, but how often do the hairy ears of the ass project above the tawny hide of the lion! Even the angels that lean over the bulwarks of heaven drop outraged tears to see the fantastic tricks played by those dressed in a little brief authority here below. There are few minor annoyances in a doctor's life more exasperating than a subpœna in which he is ordered to lay aside all other business and appear in court at a given date, and he is further admonished, not to call it threatened, to fail not under penalty of $300 fine. When one must sit in a dingy justice court, eating his heart out with impatience, wasting precious time, until he is called to give testimony in a case in which he has no manner of interest, and which is nothing more nor less than an unspeakable bore. Yet he has no redress. He must submit or pay the penalty. This may be law, but equity it is not. Should a doctor disregard the summons and persist in the performance of that which he considers his plain duty, there is no justice in the statute that would make a martyr of him. His conduct would not establish a bad precedent nor retard the machinery of the law, which seems to be the inference, but would be excusable, even commendable, from the humanitarian view. It may be said to their credit that there are many just judges who regard the situation in the proper light, but the most of them have no consideration whatever for the physician and his professional obligations if they chance to conflict with the routine work of the law. If the doctor is exempted from jury duty, we fail to understand why the same exemption should not be accorded him in the matter of testimony in open court, except in unusually important cases. His sworn depositions may be taken without interrupting him in his duties, and will be equally as effective as if delivered from the witness stand. The profession of medicine is not antagonistic to the law, quite the reverse, and it is equally sure that it is not subordinate or secondary to it in its aims and purposes as effecting the greatest good to the greatest number. We honestly think that the relation of the two professions toward each other should be established on a basis of respect for mutual rights and privileges, and not as at present on the assumption that the law dominates all else.

